# microRNA-488 inhibits chemoresistance of ovarian cancer cells by targeting Six1 and mitochondrial function

**DOI:** 10.18632/oncotarget.20941

**Published:** 2017-09-15

**Authors:** Zhuo Yang, ZiYi Feng, JiaHui Gu, XinHui Li, QianZhe Dong, KuiRan Liu, Yan Li, Ling OuYang

**Affiliations:** ^1^ Department of Obstetrics and Gynecology, Shengjing Hospital Affiliated to China Medical University, Heping District, Shenyang, Liaoning, People's Republic of China; ^2^ China Medical University, Shenbei New District, Shenyang, Liaoning, People's Republic of China; ^3^ Department of Pathology, China Medical University, Shenbei New District, Shenyang, Liaoning, People's Republic of China

**Keywords:** ovarian cancer, miR-488, Six1, mitochondrial dynamics, Drp1

## Abstract

Dysregulation of miR-488 has been implicated in several human cancers. In this study, we aim to explore its expression and biological function in ovarian cancers. We found miR-488 expression was downregulated in ovarian cancer tissues. Using CCK8 and colony formation assay showed that miR-488 inhibited SKOV3 cell proliferation and colony formation, with downregulation of cyclin D1 and cyclin E protein. While miR-488 inhibitor promoted OVCAR3 cell growth and colony formation. Cell viability and Annexin V/PI staining showed that miR-488 downregulated cell survival and increased apoptosis rate when treated with cisplatin and paclitaxel. Further experiments using MitoTracker and JC-1 staining indicated that miR-488 regulated mitochondrial fission/fusion balance and inhibited mitochondrial membrane potential, with p-Drp1, Drp1 and Fis1 downregulation. Luciferase reporter assay showed that Six1 is a target of miR-488. We also found a negative association between Six1 and miR-488 in ovarian cancer tissues. In addition, Six1 overexpression induced mitochondrial fission and increased mitochondrial potential, with upregulation of Drp1 signaling. Six1 depletion showed the opposite effects. Restoration of Six1 in SKOV3 cells rescued decreased p-Drp1 and Drp1 expression induced by miR-488 mimic. Six1 plasmid also reversed the effects of miR-488 on chemoresistance and apoptosis. Taken together, the present study showed that, by targeting Six1, miR-488 inhibits chemoresistance of ovarian cancer cells through regulation of mitochondrial function.

## INTRODUCTION

Ovarian cancer is one of the leading cause of death in women worldwide [1, 2]. Despite recent advances of combined therapies including surgery and chemotherapies, the prognosis of advanced stage ovarian cancers remains poor. In addition, the molecular mechanisms involved in ovarian carcinogenesis and chemoresistance are poorly defined, which limits the efficiency of clinical treatment. Thus identifying molecular targets which are responsible for ovarian cancer progression and drug resistance is crucial for the development of novel diagnostic and therapeutic strategies [3, 4].

microRNAs (miRNA) exert their biological function through post-transcriptional downregulation of target genes [5, 6]. Dysregulation of microRNA contributes to ovarian carcinogenesis and malignant progression [7–10]. miR-488 participates in the process of several human diseases such as peritoneal fibrosis and panic disorder [11, 12]. miR-488 dysregulation is also involved in carcinogenesis. miR-488 inhibits proliferation and induces apoptosis by targeting androgen receptor in prostate cancer [13]. miR-488 targets ZIP8 in osteoarthritis which reduced cartilage degradation [14]. miR-488 is downregulated in gastric cancers and functions as a tumor suppressor by targeting PAX6 expression [15]. To date, clinical significance of miR-488 and its biological function in ovarian cancers have not been explored.

Mitochondria plays an important role during development of chemoresistance in ovarian cancer cells. In sensitive ovarian cancer cells, it is more likely for cisplatin to cause mitochondrial dysfunction, mitochondrial release of cytochrome c and mitochondrial superoxide and hydrogen peroxide production compared with resistance cell lines [16, 17]. Thus change of mitochondrial function is important for the development of chemoresistance.

In this study, we examined miR-488 expression in paired ovarian cancer tissues using realtime PCR. We further investigated the effects and mechanisms of miR-488 on chemoresistance and mitochondrial function of ovarian cancer cells.

## RESULTS

### miR-488 is downregulated in ovarian cancers

**Figure 1:**
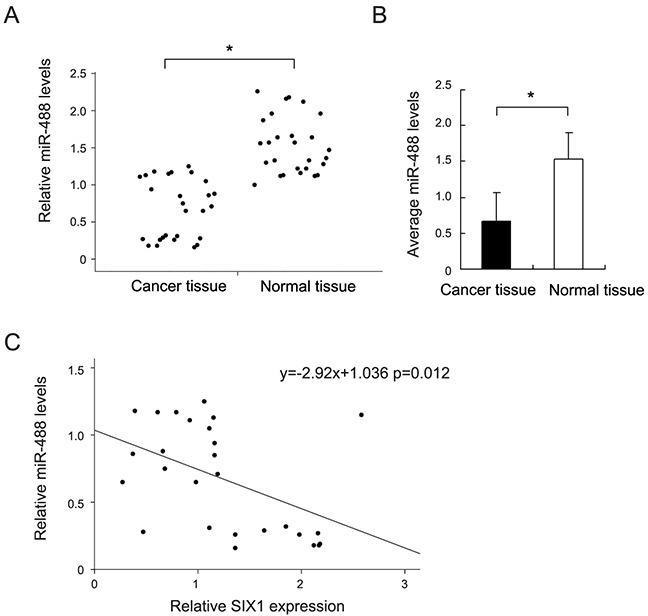
Expression pattern of miR-488 in ovarian cancer tissue samples **(A)** Relative expression level of miR-488 in 27 cases of fresh ovarian cancer tissues and paired normal tissues. **(B)** Mean miR-488 level of 27 fresh ovarian cancer tissues and corresponding normal tissues. **(C)** Correlation between miR-488 and Six1 mRNA in ovarian cancer tissues using linear correlation. *: p<0.05.

We examined miR-488 expression in 27 pair of serous ovarian carcinoma tissues with adjacent normal ovarian tissues. Mean miR-488 expression in ovarian cancers was lower than that in normal ovarian tissues (Student's t test, p<0.05) (Figure 1A&1B). miR-488 in cancer tissues/mean miR-488 value in normal tissues <2 was regarded as significant miR-488 downregulation. We found miR-488 downregulation in 11 out of 27 serous ovarian carcinoma tissues.

### miR-488 inhibits cell proliferation in ovarian cancer cells

**Figure 2:**
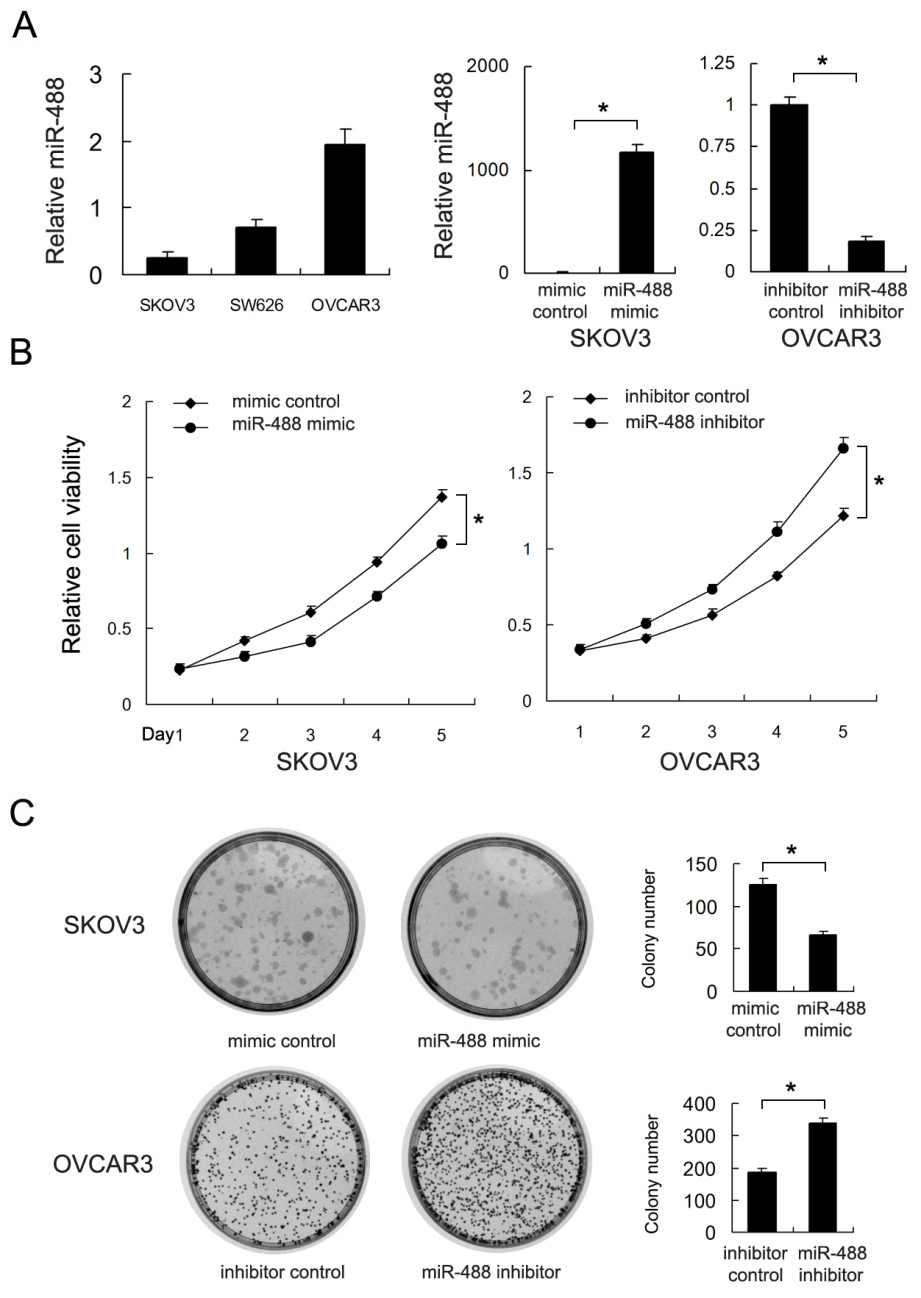
Effects of miR-488 on cell proliferation and invasion in ovarian cancer **(A)** Expression level of miR-488 in three ovarian cancer cell lines (SW626, SKOV3 and OVCAR3). miR-488 mimic upregulated miR-488 level in SKOV3 cell line and its inhibitor downregulated miR-488 level in OVCAR3 cell line. **(B)** CCK8 assay revealed that miR-488 mimic inhibited growth rate while miR-488 inhibitor accelerated growth rate. **(C)** miR-488 mimic decreased colony number of SKOV3 cells. miR-488 inhibitor increased colony number of OVCAR3 cells. *: p<0.05.

Real-time PCR was used to examine the expression of miR-488 in three ovarian cancer cell lines including SW626, SKOV3 and OVCAR3. We found that SKOV3 has lowest miR-488 expression and OVCAR3 has the highest miR-488 expression. Transfection of miR-488 mimic and inhibitor was performed in SKOV3 and OVCAR3 cell lines respectively. Transfection effects were confirmed in both cell lines (Figure 2A). By using CCK8, we found that miR-488 mimic downregulated ovarian cancer proliferation rate while miR-488 inhibitor accelerated ovarian cancer proliferation (p<0.05) (Figure 2B). Colony formation assay showed that miR-488 mimic downregulated colony number while miR-488 inhibitor increased colony number (p<0.05) (Figure 2C).

### miR-488 reduces chemoresistance in ovarian cancer cells

**Figure 3:**
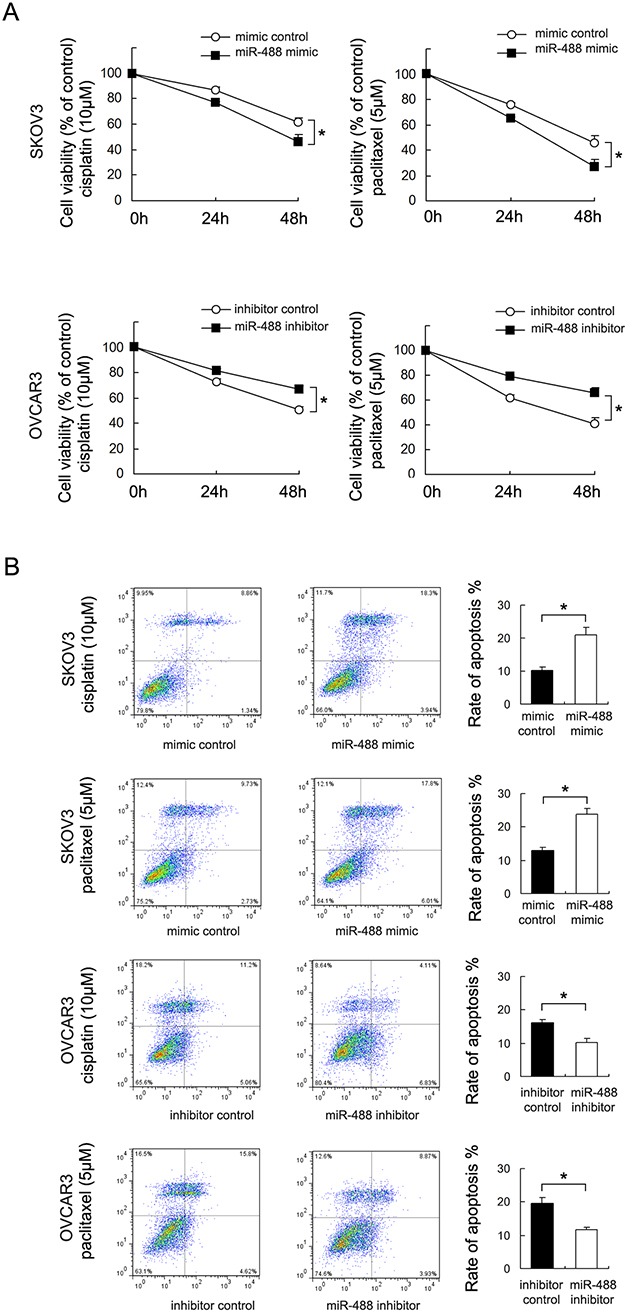
miR-488 reduces chemoresistance to cisplatin and paclitaxel **(A)** miR-488 mimic significantly downregulated SKOV3 cell viability at 48 hours of cisplatin and paclitaxel treatment. miR-488 inhibitor showed the opposite effect by upregulating cell viability. **(B)** Annexin V/PI staining showed that miR-488 mimic significant upregulated apoptosis rate in SKOV3 cells. miR-488 inhibitor downregulated apoptosis in OVCAR3 cells. *: p<0.05.

To investigate the impact of miR-488 on chemoresistance, CCK8 cell viability assay was used to examine cell survival after treatment with cisplatin and paclitaxel (Figure 3A). miR-488 mimic inhibited cell viability in SKOV3 cells after 24 and 48 hours of treatment with cisplatin (10μM) and paclitaxel (5μM). While miR-488 inhibitor conferred cisplatin and paclitaxel resistance by upregulating OVCAR3 cell viability (p<0.05).

Annexin V/PI staining was then adopted to check the level of apoptosis. As shown in Figure 3B, miR-488 mimic transfection significant upregulated apoptosis rate in SKOV3 cells treated with 24 hours of cisplatin (10μM) and paclitaxel (5μM) (p<0.05). While miR-488 inhibitor downregulated apoptosis rate in OVCAR3 cells treated with 24 hours of cisplatin (10μM) and paclitaxel (5μM) (p<0.05). These data demonstrated that miR-488 could inhibit multi-drug resistance in ovarian cancers.

### miR-488 regulates mitochondrial dynamics and membrane potential in ovarian cancer cells

**Figure 4:**
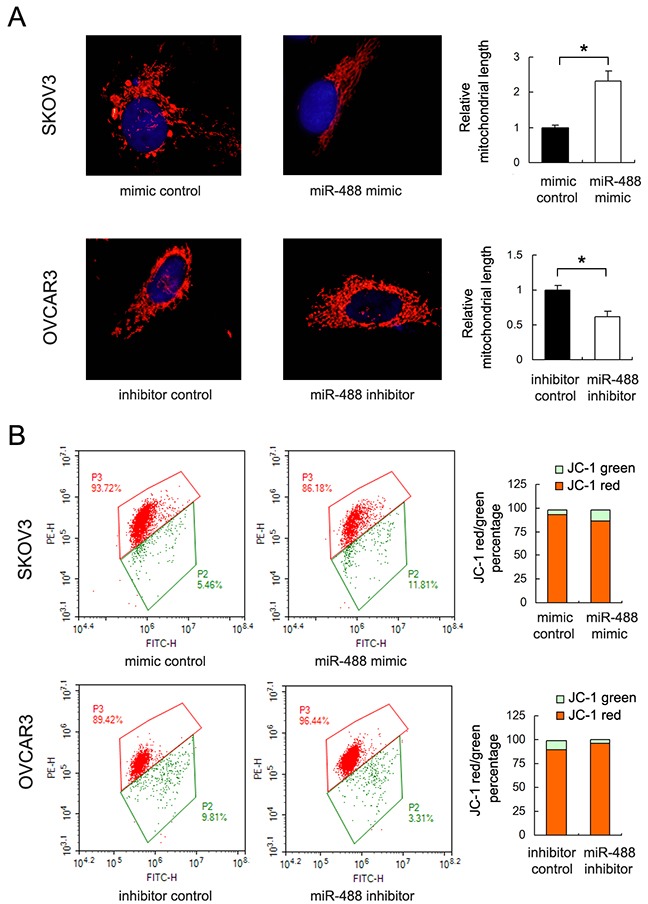
miR-488 regulates mitochondrial dynamics and membrane potential **(A)** Mitochondrial morphology was evaluated with MitoTracker Red staining. Mitochondrial shape were prone to fusion after miR-488 mimic in SKOV3 cells. miR-488 inhibitor lead to mitochondrial fission in OVCAR3 cells. **(B)** Flow cytometry showed JC-1 distribution after miR-488 mimic/inhibitor transfection. miR-488 mimic downregulated membrane potential while miR-488 inhibitor upregulated membrane potential. *: p<0.05.

Resistance to chemotherapy is frequently regulated by mitochondrial apoptosis pathway. To find out if miR-488 is involved in the mitochondrial dynamic balance, we checked mitochondrial morphology. Mitochondria in cells with miR-488 mimic was more prone to fusion with elongated mitochondrial shape in SKOV3 cells. While in OVCAR3 cells transfected with miR-488 inhibitor, mitochondria developed to fission with shorter mitochondrial shape (p<0.05) (Figure 4A).

Then we assessed if miR-488 was able to control mitochondrial apoptosis by regulating mitochondrial membrane potential. Quantitative analysis was carried out using flow cytometry. In cells with low membrane potential, JC-1 showed green fluorescence instead of red. Our results showed that, in SKOV3 cells with miR-488 mimic, the percentage of red fluorescence decreased compared with control, indicating decreased mitochondrial membrane potential. While in OVCAR3 cells with miR-488 inhibitor, the percentage of red fluorescence was upregulated. These data indicated that miR-488 is a negative regulator of mitochondrial membrane potential.

### miR-488 regulates cell cycle proteins and Drp1 signaling

**Figure 5:**
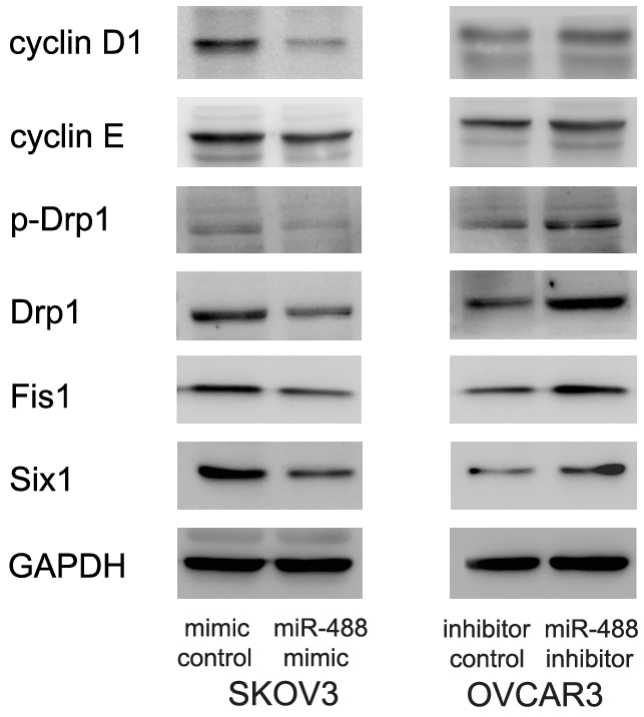
miR-488 regulates cell cycle proteins and Drp1 phosphorylation Western blot displayed that cyclin D1, cyclin E, p-Drp1, Drp1, Fis1 and Six1 of SKOV3 cells were decreased with miR-488 mimic treatment. cyclin D1, cyclin E, p-Drp1, Drp1, Fis1 and Six1 of OVCAR3 cells were upregulated after transfection of miR-488 inhibitor.

We checked cell cycle related proteins. miR-488 mimic decreased cyclin D1, cyclin E protein expression while miR-488 inhibitor upregulated cyclin D1 and cyclin E (Figure 5).

Since miR-488 regulates mitochondrial function and dynamics, we examined change of mitochondrial regulators including p-Drp1, Drp1 and Fis1. We discovered that miR-488 mimic downregulated the protein levels of p-Drp1, Drp1 and Fis1, while miR-488 inhibitor showed the opposite effect on these proteins (Figure 5).

### miR-488 targets and downregulates Six1 in ovarian cancer cells

**Figure 6:**
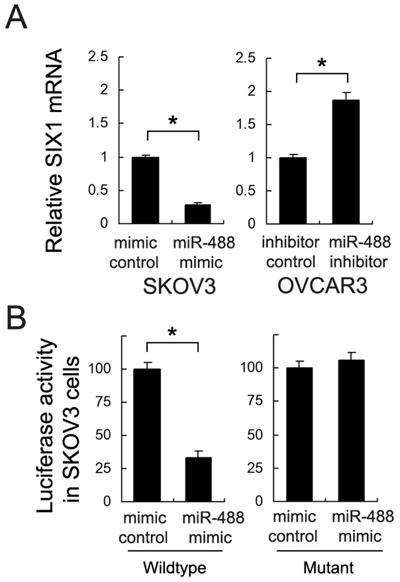
Six1 is a direct target of miR-488 in ovarian cancer cells **(A)** miR-488 mimic inhibited mRNA expression of Six1 while miR-488 inhibitor upregulated the mRNA expression of Six1. **(B)** Luciferase reporter plasmid with wild-type and mutant binding site was transfected into SKOV3 cells with miR-488 mimic. When transfected with wild-type reporter, cells showed a downregulated luciferase activity with miR-488 mimic. When transfected with mutant site reporter, there was no significant change. *: p<0.05.

We screened and examined potential targets of miR-488 using TargetScan 7.0. We found that miR-488 mimic downregulated Six1 while miR-488 inhibitor increased Six1 expression at both protein and mRNA levels (p<0.05) (Figures 5&6A).

To validate the relationship between miR-488 and Six1, we used luciferase reporter assay. Luciferase reporter is designed to quantitatively evaluate miRNA activity by the insertion of miRNA target sites downstream of the firefly luciferase gene. Reduced firefly luciferase expression indicates the binding of introduced miRNAs to the cloned miRNA target sequence. Wild-type (CUUUCA) and mutant (CAAACA) were cloned into reporter. miR-488 mimic downregulated reporter activity in SKOV3 cells after transfection of wild-type vector (p<0.05) (Figure 6B). We did not found significant changes in cells transfected with mutant vector, indicating miR-488 binds to Six1 3′-UTR and suppresses its expression. Furthermore, we checked the association between miR-488 and Six1 mRNA in 27 cases of serous ovarian cancers. A negative association was found between miR-488 and Six1 (Linear regression model, p<0.05, Figure 1C).

### Six1 modulates mitochondrial fusion/fission balance and maintains mitochondrial membrane potential

**Figure 7:**
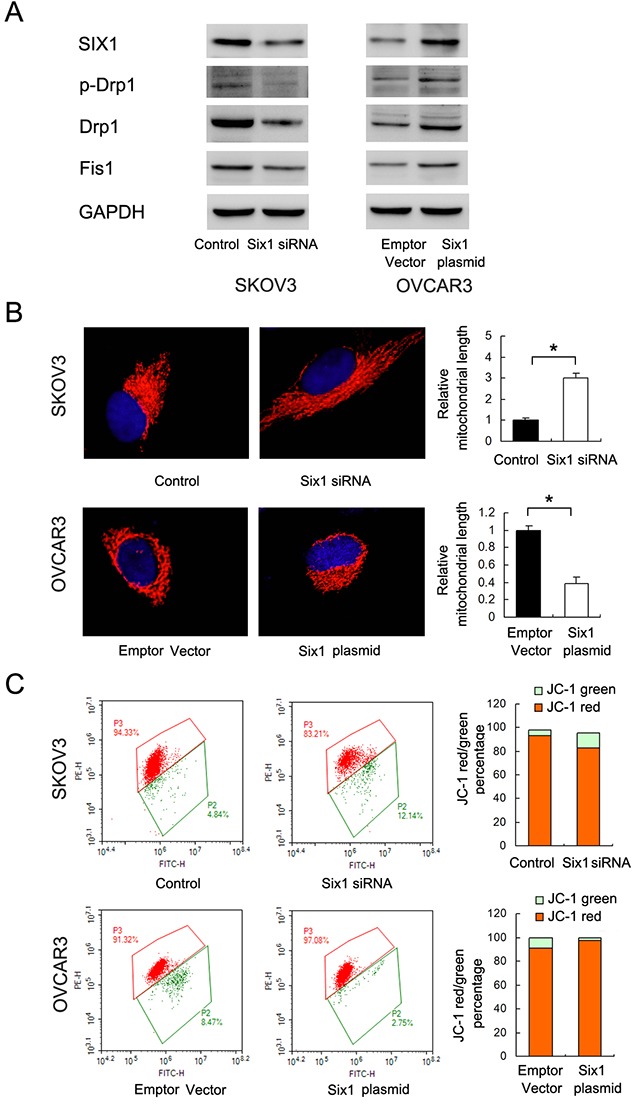
Six1 induces mitochondrial fission and maintains mitochondrial membrane potential **(A)** Six1 overexpression upregulated p-Drp1, Drp1, Fis1 in OVCAR3 cells while Six1 depletion downregulated p-Drp1, Drp1, Fis1 in SKOV3 cells. **(B)** Six1 overexpression induced mitochondrial fission while its depletion induced mitochondrial fusion. **(C)** Flow cytometry showed Six1 plasmid downregulated JC-1 green cell population, which indicated that Six1 positively regulated mitochondrial membrane potential. *: p<0.05.

Then we examined Six1 function using plasmid/siRNA transfection. As shown in Figure 7A&7B, Six1 depletion induced mitochondrial fusion with downregulation of p-Drp1, Drp1 and Fis1 in SKOV3 cells. Six1 overexpression in OVCAR3 cells induced mitochondrial fission and upregulated p-Drp1, Drp1 and Fis1. JC-1 staining demonstrated that Six1 increased mitochondrial membrane potential while its depletion reduced mitochondrial membrane potential (Figure 7C). Together these results demonstrated that Six1 is able to induce mitochondrial fission and maintain mitochondrial membrane potential, which reduced sensitivity of cancer cells to mitochondrial apoptosis.

### Six1 restores Drp1 signaling which is reduced by miR-488

**Figure 8:**
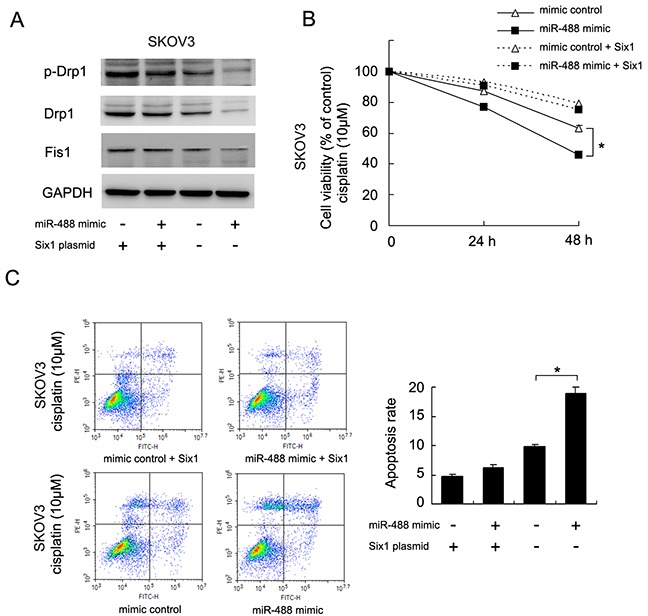
miR-488 regulates Drp1 signaling and chemoresistance through Six1 **(A)** Western blot revealed that miR-488 mimic could inhibited p-Drp1 and Drp1. In Six1 overexpressed cells, treatment with miR-488 mimic failed to reduce p-Drp1, Drp1 and Fis1 levels. **(B)** CCK8 assay demonstrated that Six1 overexpression restored cisplatin resistance which was downregulated by miR-488. **(C)** Annexin V/PI staining showed that Six1 overexpression abrogate the apoptosis inducing effect of miR-488. *: p<0.05.

To confirm the involvement of Six1 in miR-488 mediated regulation of Drp1 signaling, we overexpressed Six1 in SKOV3 cells treated with miR-488 mimic. Western blot revealed that miR-488 mimic could inhibited p-Drp1, Fis1 and Drp1. However, in Six1 overexpressed cells, the effects of miR-488 mimic on p-Drp1, Drp1 and Fis1 were not significant. Six1 overexpression also restored p-Drp1, Drp1 and Fis1 levels which were downregualted by miR-488 (Figure 8A). CCK8 assay demonstrated that Six1 overexpression also restored cisplatin resistance which was downregulated by miR-488 (Figure 8B). Annexin V/PI staining showed that Six1 overexpression abrogate the apoptosis inducing effect of miR-488 (Figure 8C). These data together demonstrated that Six1 plays a central role in miR-488 induced change of chemosensitivity and mitochondrial function.

## DISCUSSION

Growing evidences demonstrate the roles of miRNAs during ovarian carcinogenesis [18–20]. miR-488 was reported to function as a tumor suppressor in human prostate and gastric cancer by targeting androgen receptor and PAX6 [13, 15]. Nevertheless, expression pattern and roles of miR-488 in human ovarian cancer remains unclear. Our data showed that miR-488 expression was lower in ovarian cancer tissues compared with normal tissues, which was in accord with previous reports, indicating miR-488 is a potential tumor suppressor in ovarian cancers [15, 21].

CCK8 and colony formation assay showed that miR-488 downregulated cell growth rate and colony formation ability. Accordingly, cyclin D1 and cyclin E protein were downregulated. cyclin D1 and cyclin E are cell cycle regulators which phosphorylated Rb to enable G1-S cell cycle transition [22–25]. These data indicate that miR-488 negatively regulates ovarian cancer growth through inhibition of cell cycle transition.

Next we checked the role of miR-488 on drug resistance and apoptosis. Our results showed that miR-488 significantly reduced cisplatin and paclitaxel resistance, with upregulated apoptosis rate. Chemotherapeutic drug, especially platinum-containing agents, could induce cancer apoptosis through mitochondrial apoptosis pathway. So we checked if miR-488 was able to regulate mitochondrial apoptosis by changing mitochondrial function, which includes mitochondrial dynamics and mitochondrial membrane potential.

Mitochondrial dynamics is a balance between fusion and fission, which could be modulated by Drp1 phosphorylation [26]. The effect of mitochondrial dynamics on chemoresistance is dependent on cell type and microenvironment. Mitochondrial fission was reported to promote cancer growth and inhibit mitochondrial apoptosis. While mitochondrial fusion makes cancer cells prone to mitochondrial apoptosis [27]. Our results showed that miR-488 induced mitochondrial fusion, with downregulation of p-Drp1 and Fis1. Drp1 phosphorylation and Fis1 upregulation promotes their mitochondrial membrane localization and induces mitochondrial fission [28], which was in accord with fragmented mitochondrial shape. Drp1-dependent mitochondrial dynamics may confer either apoptosis or resistance. In normal cells, mitochondrial fission is required for Bax -dependent cytochrome c release from mitochondria [29]. Inhibition of mitochondrial fission by Drp1 depletion or inhibitor blocked cell cycle and resulted in apoptosis in lung and colon cancer cells [30, 31]. mdivi-1 sensitized chemoresistant breast and lung cancer cells to CDDP [32]. Mitochondrial fission has been shown to repress respiratory complex I and IV. Since respiratory complex I and -III are the main sources of ROS, repression of complex I by fission provides an explanation for resistance to several forms of mitochondrial apoptosis induced by ROS. ROS production could lead to impaired mitochondrial membrane potential.

We also examined mitochondrial membrane potential using JC-1 staining. We found that miR-488 mimic reduced membrane potential while its inhibitor maintained membrane potential. Loss of membrane potential triggers mitochondrial apoptosis pathway through elevated mitochondrial membrane permeability and release of cytochrome c. Thus our data identified miR-488 as a negative regulator of mitochondrial function, which reduces resistance to mitochondrial apoptosis in ovarian cancer cells.

TargetScan software was used to predict potential miR-488 target and Six1 was on the target list. Six1 is an oncoprotein which was overexpressed in ovarian cancer [33]. Our data demonstrated that miR-488 is a negative regulator of Six1 mRNA and protein. Binding of to Six13′-UTR was further confirmed by Luciferase reporter assay. In addition, we found a negative correlation between miR-488 and Six1 mRNA in ovarian cancer tissues, which further strengthen the link between them.

Six1 functions as an oncoprotein in ovarian cancer [33]. Six1 induces resistance to TRAIL mediated apoptosis in ovarian cancer cells [34]. Six1 also mediates resistance to paclitaxel in breast cancer cells [35]. A recent report showed that Six 1 inhibits mitochondrial apoptosis pathway via caspase-7 in gastric cancer cells [36], suggesting a potential link between Six1 and mitochondrial function in human cancers. Six1 also induces radioresistance via AKT/Bcl-2 pathway in esophageal squamous cell carcinoma and Bcl-2 is an inhibitor of mitochondrial apoptosis [37]. These studies did not investigate the association between Six1 and mitochondrial dynamics. In this study, we found that Six1 is a positive regulator of mitochondrial fission and Drp1 phosphorylation. We also demonstrated that Six1 could maintain mitochondrial membrane potential in ovarian cancer cells. These data indicated that Six1 might serve as the mediator of miR-488 induced chemosensitivity. Six1 has been reported to activate ERK signaling in several cancers [38, 39]. It has been reported that activation of ERK signaling could lead to Drp-1 phosphorylation and mitochondrial fission, which is involved in the drug resistance of leukemia cells [28]. Thus Six1 may induce mitochondrial fission through ERK/Drp1 signaling.

To further validate the role of Six1 in miR-488 induced chemosensitivity, we adopted Six1 plasmid to restore its expression in miR-488 treated SKOV3 cells. Six1 plasmid restored Drp1 and p-Drp1 status downregulated by miR-488. Six1 also restored chemoresistance in ovarian cancer cells, which was reduced by miR-488 mimic. Taken together, our data demonstrated miR-488 inhibits chemoresistance in ovarian cancer through downregulation of Six1.

In conclusion, this study demonstrated that miR-488 targets and downregulates Six1 and subsequently inhibits Drp1 signaling to regulate mitochondrial function and chemoresistance. Our results provide insight into a novel biomarker which predict ovarian cancer chemosensitivity.

## MATERIALS AND METHODS

### Patients and specimens

This study was performed with the approval of the Ethics Committee and Institutional Review Board of Shengjing Hospital. All patients provided written informed consent for their data to be used in the study. Fresh ovarian cancer specimens and corresponding normal tissues were collected from patients from 2011 to 2015 with informed consent. Tissues were stored at -80°C before RNA extraction.

### Cell culture and transfection

SW626, SKOV3 and OVCAR3 cell lines were obtained from American Type Culture Collection (Manassas, USA). Cells were cultured in DMEM (Gibco, USA) withy 10% fetal bovine serum (Invitrogen, Carlsbad, CA, USA). miR-488 mimic/miR-488 inhibitor and corresponding controls were obtained from RiboBio (Guangzhou, China). Six1 siRNA and Negative control siRNA were purchased from Dharmacon (GE healthcare, USA). *T*ransfection of miRNA and siRNA was performed with DharmaFECT transfection reagent (GE, USA). pCMV6-Six1 construct was purchased from Origene company (Origene, USA). Lipofectamine 3000 (Life technology, USA) was used for transfection.

### Realtime PCR

RNA extraction was performed using RNAiso (TAKARA, China). Realtime PCR was performed using SYBR Green Master Mix from ABI (Applied Biosystem, USA). Realtime PCR was carried out using ABI 7500 (Applied Biosystems, USA). Relative quatification of target genes was calculated using the 2^−ΔΔCt^ method. miR-488 and U6 primers were obtained from RiboBio (RiboBio, Guangzhou, China). The primer sequences for Six1 and actin are listed as followers: Six1 forward, 5′ AAGGAG AAGTCGAGGGGTGT 3′, Six1 reverse, 5′ TGCTTGTT GGAGGAGGAGTT 3′; β-actin forward, 5′-ATAGCACAGCCTGGATAGCAACGTAC-3′; β-actin reverse, 5′-CACCTTCTACAATGAGCTGCGTGTG-3′.

### Western blot

After cell lysis, protein quantification was performed using Bradford method. 30 μg protein was transferred to PVDF membranes after separated by SDS-PAGE. PVDF membrane was incubated with the following antibodies: Six1 (1:800, Sigma), cyclinE, cyclin D1, p-Drp1, Drp1, Fis1, and GAPDH (1:1000, Cell Signaling Technology, USA). Then membranes were incubated with HRP-coupled anti-mouse/rabbit IgG (1:2000, Cell Signaling Technology, USA) at 37°C for 2 hours. After that protein bands on the membranes were visualized using ECL kit (Thermo Fisher, IL, USA) with DNR BioImaging System (DNR, Israel).

### CCK8 assay and colony formation

To perform colony formation, cells were seeded in culture dishes and cultured for 2 weeks. Then these plates were stained using Giemsa. We performed CCK8assay with Cell Counting Kit-8 kit (Dojindo) with a plate reader. 96 well cell plate was examined at 490 nm.

### Annexin V/PI analysis

Annexin V/PI Kit (BD bioscience, USA) was used to examine apoptosis. After treatment, cells were harvested by 0.25% trypsin, washed with PBS and resuspended in binding buffer. Staining solution containing Annexin V/FITC and propidium iodide (PI) was added in cell suspension. After incubation in the dark for 30 minutes, rate of apoptosis was analyzed by ACEA flow cytometer.

### Target gene validation and luciferase reporter assay

We use luciferase reporter vector to validate the association between miR-488 and Six1. We adopted luciferase reporter vector with wild-type miR-488 binding site at Six1 3′-UTR CUUUCA (2119-2125 of Six1 3′ UTR). We also used mutant miR-488 binding site CAAACA.

Cells were co-transfected with wild-type and mutant luciferase reporter along with the Renilla luciferase reporter, which was then measured using dual luciferase reporter gene assay kit (Promega, CA, USA).

### Mitochondrial membrane potential

The mitochondrial membrane potential (Δψm) was detected by using JC-1 staining method. Briefly, cells were harvested, washed with PBS and incubated with 5 μM JC-1 (Cell Signaling Technology) for 30 minutes in the incubator. Then cells were washed and analyzed using a ACEA flow cytometer (ACEA, USA). Data was analyzed using Novoexpress software (ACEA, USA).

### Immunofluorescence of mitochondria

Mitochondria in cancer cells was stained using MitoTracker red (Life Technologies) for 30 minutes. Nucleus was stained using Hoechst 33258. Images were taken by Olympus BX53 microscope. The length of mitochondrial was examined by ImageJ software.

### Statistical analysis

SPSS 17 was used for analysis. t-test was used to compare data. p<0.05 was regarded as statistical significant.

## Abbreviations

CCK8: Cell Counting Kit-8
ROS: reactive oxygen species
